# Renal-type clear cell carcinoma of the prostate: a diagnostic challenge

**DOI:** 10.1186/s13000-015-0432-8

**Published:** 2015-10-24

**Authors:** Shashikant C. U. Patne, Nidhi Johri, Richa Katiyar, Sameer Trivedi, Uday Shankar Dwivedi

**Affiliations:** Department of Pathology, Institute of Medical Sciences, Banaras Hindu University, Varanasi, 221 005 UP India; Department of Urology, Institute of Medical Sciences, Banaras Hindu University, Varanasi, 221 005 UP India

**Keywords:** Renal cell carcinoma, Male, Prostate-specific antigen, Prostatectomy, Prostatic neoplasms

## Abstract

A 72-year-old male presented with urinary symptoms. His serum prostate specific antigen level was 65.2 ng/ml. His radical prostatectomy specimen showed clear cell lesion reminiscent of the clear cell renal cell carcinoma along with acinar type of prostatic adenocarcinoma, Gleason score 4 + 4. The lesional clear cells were positive for pancytokeratin, epithelial membrane antigen, CD10, vimentin, and AMACR while negative for 34βE12, CK7, prostate specific antigen, and PAX8. The final diagnosis was renal-type clear cell carcinoma of the prostate. A follow-up of 20 months did not show metastasis. We herein report fifth case of renal-type clear cell carcinoma of the prostate.

## Background

Occurrence of clear cell lesions in the prostate is rare and diagnostically challenging with variety of possible diagnoses. These include clear cell variants of commonly seen prostatic adenocarcinoma and transitional cell carcinoma together with uncommonly encountered clear cell carcinoma of mullerian type, metastatic renal cell carcinoma, and a recently described entity, renal-type clear cell carcinoma (RTCCC) of the prostate [1, 2]. RTCCC occurring in the prostate is an extremely unusual malignant tumor with only four previously published case reports [2–5]. We herein report fifth case of RTCCC of the prostate and review pertinent literature.

## Case presentation

**Fig. 1:**
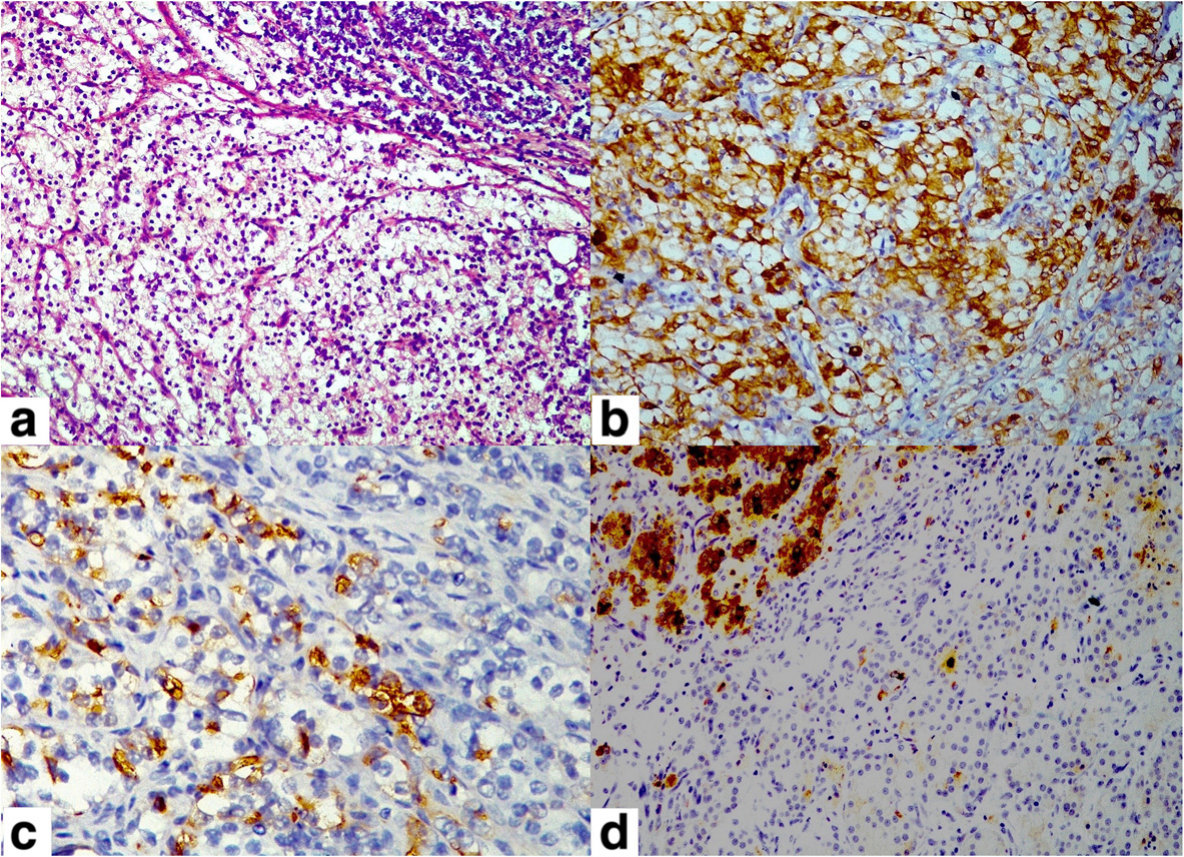
Microphotographs of the lesion. **a** Clear cell carcinoma very similar to clear cell type of renal cell carcinoma with usual prostatic adenocarcinoma on upper right corner of the photograph (Hematoxylin and eosin stain, 200×). **b** The lesional clear cells show immunostaining for pancytokeratin (Diaminobenzidine, 200×). **c** The clear cells show CD 10 immunoreactivity (Diaminobenzidine, 400×). **d** The lesional clear cells are negative for PSA immunostaining. Note that usual prostatic adenocarcinoma seen in upper left corner of the photograph show positive PSA immunostaining (Diaminobenzidine, 200×)

A 72-year-old man came to Urology OPD for evaluation of urinary frequency, urgency, and difficulty. His rectal examination revealed grade II enlargement of the prostate that was firm on palpation. His urine examination was positive for occult blood and 50–70 pus cells/high power fields. His serum prostate specific antigen (PSA) level was 65.2 ng/ml (normal <4 ng/ml). Cystoscopy of the patient showed edematous prostatic urethra and occluding lateral and median lobes of the prostate. Ultrasound and magnetic resonance imaging (MRI) examination showed enlarged prostate without any obvious abnormally enhancing mass lesion in the prostatic parenchyma, and unremarkable appearance of bilateral kidneys, urinary bladder, urethra and rest of the pelvic organs. Needle core biopsy of the prostate revealed adenocarcinoma. His 99mTc-methylene diphosphonate (MDP) scans were negative for bony metastasis. The patient underwent radical prostatectomy and bilateral pelvic lymph node dissection. Grossly, the prostate was 5.5 × 4.5 × 3.5 cm in size, cut surface revealed solid grey white areas occupying whole of the prostate except for a thin rim of normal prostate at periphery. Entire prostate gland was embedded in paraffin blocks. Microscopy showed infiltration of the prostatic parenchyma by confluent nests of epithelial cells reminiscent of clear cell carcinoma of renal origin. The epithelial cells showed enlarged centrally located nuclei, conspicuous nucleoli, and abundant clear cytoplasm (Fig. 1a). The tumor nests were surrounded by fibrovascular septa that contained lymphocytic infiltrates. Cytoplasm of few of the clear cells contained periodic acid Schiff positive droplets. Additionally, multiple foci of usual prostatic adenocarcinoma of acinar type (Gleason’s score 4 + 4) were present. With standard immunohistochemical procedure on paraffin sections, the clear cells were immunoreactive for cytokeratin cocktail (Fig. 1b), CD10 (Fig. 1c), and epithelial membrane antigen. Immunoreactivity for PSA was seen in the areas of usual prostatic acinar adenocarcinoma, but negative in the clear cell areas (Fig. 1d). The clear cells did not show immunoreactivity against high molecular weight cytokeratin (HMWCK) 34βE12, and CK7. Tumor infiltration was present in all blocks of the prostate from apex to base. Bilateral vas deferens and seminal vesicles were free of tumor. Initially, we thought of metastasis from clear cell carcinoma of renal origin. Subsequently, pre-operative MRI scans were reviewed, which did not reveal any lesion in kidney, urinary bladder, or urethra. Additional immunohistochemistry was performed, which revealed immunoreactivity of the clear cells for vimentin and alpha-methylacyl-CoA racemase (AMACR), and negative immunostaining for PAX8. After extensive literature search and review of histopathology slides, the lesion was diagnosed as RTCCC of the prostate. The patient did not give any history of receiving hormone therapy prior to surgery. There was no history or radiological evidence suggestive of von Hippel-Lindau disease in the patient. Serial measurement of serum PSA level on follow-up showed increasing levels, which reached to 11.3 ng/ml, 15-months after radical prostatectomy. The patient was given post-operative hormone therapy, after which his PSA levels started declining. On last follow-up, 20-months after the radical prostatectomy, he was doing well with serum PSA level of 0.53 ng/ml.

This case represented a diagnostic challenge because of close resemblance with clear cell renal cell carcinoma (RCC) on histology. Clear cell type is the most common variant of RCC, which shows tubules and solid nests/sheets of clear cells (atypical enlarged nuclei with prominent nucleoli) with surrounding richly vascularized stroma containing interstitial inflammatory cells [2]. RCC demonstrates immunopositivity for low molecular weight cytokeratin and vimentin, while it is negative for HMWCK, CK7, CK20, PSA and CEA [2]. In addition, most of the clear cell RCCs are positive for epithelial membrane antigen and CD10 [6].

RTCCC of the prostate is a novel entity, which was first reported by Singh *et al*. [2]*.* Our literature search has revealed only four previously published case-reports of RTCCC of the prostate, which are given in Table 1. Histopathological and immunohistochemical features of RTCCC of the prostate resemble with the clear cell RCC [2–5]. In such a situation, it becomes difficult for pathologists to rule out metastasis of clear cell RCC to the prostate. However, to our knowledge, metastasis of RCC to the prostate at initial diagnosis has not been reported. Metastatic involvement of the prostate by primary RCC has been reported in only two cases [7, 8]. Such a metastasis of RCC also presents with detectable lesion in the kidney as well as metastasis involving the lung and the bone. In our case, ultrasound and MRI examinations did not reveal any lesion in the kidney along with the unremarkable appearances of urinary bladder and urethra. MDP bone scan did not reveal skeletal metastasis. Markedly raised level of serum PSA indicated prostatic origin of the malignancy, which was later confirmed by immunohistochemistry. Clear cell lesion in the present case demonstrated an immunohistochemical profile almost identical to that of RCC, with positive expression of CD10, EMA, and vimentin and a negative result for HMWCK, CK7 and PSA, which is similar to the previously described studies on RTCCC of the prostate [2–5].

**Table 1:** Cases of renal-type clear cell carcinoma of the prostate in literature

S.No.	Author (Year)[Ref]	Age (years)	Serum PSA at diagnosis	Type of specimen	Microscopy	Special stain	Immunohistochemistry	Outcome (Follow-up duration)
1.	Singh et al. (2003) [2]	73	1.5 mg/dl	TURP followed by radical cystoprostatectomy + pelvic lymph node dissection	Clear cell carcinoma similar to RCC and typical prostatic adenocarcinoma (Gleason’s score 3 + 3)	Mucicarmine staining for intracytoplasmic mucin: negative	Positive: Vimentin, EMA, LMWCK and CD10	No evidence of disease (7 months)
							Negative: PSA, PSAP, S-100, HMB-45, Broad spectrum cytokeratin (AE1/AE3), CK7, CK20, HMWCK, CA-125, CEA, ER and PR	
2.	Pal & Chowdhury (2007) [3]	64	2.1 ng/L	TURP	Clear cell carcinoma similar to RCC	Mucicarmine staining for intracytoplasmic mucin : negative	Positive: Pancytokeratin, Vimentin and EMA	No evidence of pelvic lymph node metastasis (12 months)
							Negative: PSA, PSAP and HMWCK	
3.	Permi et al. (2011) [4]	64	82 ng/ml	TURP followed by radical cystoprostatectomy + pelvic lymph node dissection	Clear cell carcinoma similar to RCC	Not done	Not done	No evidence of recurrence (12 months)
4.	Wang et al. (2015) [5]	64	10.2 ng/ml	TURP	Clear cell carcinoma similar to RCC and typical prostatic adenocarcinoma (Gleason’s score 4 + 4)	Not done	Positive: LMWCK, Vimentin, EMA, CD10, PSAP and AMACR	Death due to multiorgan failure (6 months)
							Negative: HMWCK, CEA, Broad spectrum cytokeratin and PAX8	
5.	Present case (2015)	72	65.2 ng/ml	Radical prostatectomy and pelvic lymphadenectomy	Clear cell carcinoma similar to RCC and typical prostatic acinar adenocarcinoma (Gleason’s score 4 + 4)	Periodic acid Schiff for intracytoplasmic droplets: Positive	Positive: Pancytokeratin, EMA, CD10, Vimentin and AMACR	Alive with controlled serum PSA levels (20 months)
							Negative: HMWCK (34βE12), CK7, PSA and PAX8	

*Ref* Reference in the text, *PSA* prostate specific antigen, *TURP* Transurethral resection of prostate, *RCC* renal cell carcinoma, *EMA* epithelial membrane antigen, *LMWCK* low molecular weight cytokeratin, *PSAP* prostate specific acid phosphatase, *HMWCK* high molecular weight cytokeratin, *CEA* carcinoembryonic antigen, *ER* estrogen receptor, *PR* progesterone receptor, *AMACR* alpha-methylacyl-CoA racemase

RTCCC of the prostate has been seen in patients of age 60 years and above. This recently described entity may show elevation of serum PSA, which is due to co-existing areas of typical prostatic acinar adenocarcinoma. In our case, immunoreactivity of PSA was seen in typical prostatic acinar adenocarcinoma but not in the clear cells. However, the clear cells did express AMACR (P504S), which is a marker for prostatic origin. Management of RTCCC of the prostate is same as that of prostatic adenocarcinoma; however, its biological significance and prognosis is not clear until the date. Death has occurred in a case of RTCCC of the prostate due to multi-organ failure, after 6-months of surgery.

## Conclusions

Clear cell carcinoma resembling with clear cell renal carcinoma in the prostate indicates diagnosis of renal-type clear cell carcinoma. Imaging studies, serum PSA levels, and immunohistochemistry is essential for the diagnosis. RTCCC of the prostate is a rare and novel pathological entity, which shares histological and immunohistochemical similarity with clear cell RCC. Further research is required to clarify pathogenic mechanisms and biological behavior of this unique tumor.

## Consent

Written informed consent was obtained from the patient for publication of this case report and any accompanying images. A copy of the written consent is available for review by the Editor-in-Chief of this journal.

## Abbreviations

AMACR: Alpha-methylacyl-CoA racemase
CEA: Carcinoembryonic antigen
CK20: Cytokeratin 20
CK7: Cytokeratin 7
EMA: Epithelial membrane antigen
ER: Estrogen receptor
HMWCK: High-molecular weight cytokeratin
LMWCK: Low molecular weight cytokeratin
MDP: 99mTc-methylene diphosphonate
MRI: Magnetic resonance imaging
PR: Progesterone receptor
PSA: Prostate-specific antigen
PSAP: Prostate specific acid phosphatase
RCC: Renal cell carcinoma
RTCCC: Renal-type clear cell carcinoma
TURP: Transurethral resection of prostate

## References

[1] Humphrey PA (1997). Clear cell neoplasms of the urinary tract and male reproductive system. Semin Diagn Pathol.

[2] Singh H, Flores-Sandoval N, Abrams J (2003). Renal-type clear cell carcinoma occurring in the prostate. Am J Surg Pathol.

[3] Pal DK, Chowdhury MK (2007). Renal type clear cell carcinoma of prostate. Indian J Surg.

[4] Permi H, Laxminarayana KP, Yeshvanth S, Shetty J (2011). Renal type clear cell carcinoma of the prostate: a diagnostic dilemma. J Lab Physicians.

[5] Wang Q, Xue Y (2015). Renal-type clear cell carcinoma of the prostate: a case report. Oncol Lett.

[6] Sangoi AR, Fujiwara M, West RB, Montgomery KD, Bonventre JV, Higgins JP (2011). Immunohistochemical distinction of primary adrenal cortical lesions from metastatic clear cell renal cell carcinoma: a study of 248 cases. Am J Surg Pathol.

[7] Cihak RW, Haas R, Koenen CT, Chinchinian H (1980). Metastatic renal carcinoma to the prostate gland: presentation as prostatic hypertrophy. J Urol.

[8] King DH, Centeno AS, Saldivar VA, Sarosdy MF (1995). Renal cell carcinoma metastatic to the gallbladder or prostate: two case reports. Urology.

